# How to Accelerate Early Stage of Malaria Vaccine Development by Optimizing Functional Assays

**DOI:** 10.3390/vaccines12060586

**Published:** 2024-05-28

**Authors:** Kazutoyo Miura

**Affiliations:** Laboratory of Malaria and Vector Research, National Institute of Allergy and Infectious Diseases, National Institutes of Health, Rockville, MD 20852, USA; kmiura@niaid.nih.gov

**Keywords:** malaria, *Plasmodium falciparum*, vaccine, assay, correlate of protection, assay qualification, assay validation

## Abstract

While two *Plasmodium falciparum* circumsporozoite protein-based pre-erythrocytic vaccines (PEV), RTS,S and R21, have been approved by the WHO, no blood-stage vaccine (BSV) or transmission-blocking vaccine (TBV) has reached a phase 3 trial. One of the major obstacles that slows down malaria vaccine development is the shortage (or lack) of in vitro assays or animal models by which investigators can reasonably select the best vaccine formulation (e.g., antigen, adjuvant, or platform) and/or immunization strategy (e.g., interval of inoculation or route of immunization) before a human phase 2 trial. In the case of PEV, RTS,S and R21 have set a benchmark, and a new vaccine can be compared with (one of) the approved PEV directly in preclinical or early clinical studies. However, such an approach cannot be utilized for BSV or TBV development at this moment. The focus of this review is in vitro assays or in vivo models that can be used for *P. falciparum* BSV or TBV development, and I discuss important considerations during assay selection, standardization, qualification, validation, and interpretation of the assay results. Establishment of a robust assay/model with proper interpretation of the results is the one of key elements to accelerate future vaccine development.

## 1. Introduction

Malaria morbidity and mortality, the majority of which are caused by *Plasmodium falciparum* infections, are still unacceptably high, and there were 249 million malaria cases and 608,000 malaria-related deaths globally in 2022 according to the World Health Organization (WHO) [1]. To fight against this deadly infectious disease, the WHO recently recommended two *P. falciparum* circumsporozoite protein (PfCSP)-based pre-erythrocytic vaccines (PEV), RTS,S/AS01 (RTS,S) and R21/Matrix-M (R21) [1]. A large-scale implementation of the two vaccines in multiple African countries started in 2024, and the two vaccines are expected to have a significant impact on malaria control [2,3]. A PEV is designed to block parasite infection in human hosts; thus, it can prevent all subsequent symptoms and transmission if it works perfectly. However, if one parasite escapes from the immunity, it can proliferate normally in the vaccinee, then transmit malaria to next hosts, unless there is another mechanism to block asexual and sexual parasite growth. Therefore, the search for additive tools, such as development of other classes of vaccines, blood-stage vaccine (BSV), or transmission-blocking vaccine (TBV), must be continued. 

To make a BSV or TBV, the clinical efficacy needs to be assessed in a human phase 2 (Ph2) trial and eventually in a phase 3 (Ph3) trial, unless a “correlate of protection” (CoP) has been established based on the preceding studies with the same or very similar vaccines. However, before reaching a human phase 1 (Ph1) trial, it is very common to select “the best” vaccine formulation(s) (e.g., antigen or antigen combination, adjuvant, and vaccine platform) and/or immunization strategy(s) (e.g., interval of inoculation or route of immunization) using an in vitro assay(s) with samples collected from a preclinical animal immunization study(s) or using an in vivo animal model(s). For simplicity, hereafter, “assay” or “assays” terminologies in this review include both “in vitro assays” and “in vivo or ex vivo animal models”. Not only preclinical samples but also samples from a Ph 1 human trial are usually evaluated by the same assay(s) to select a vaccine formulation/strategy for the next Ph 2 trial. Therefore, establishment of a robust assay and of a proper analytical method is one of the critical elements for vaccine development. If a bad decision is made due to the poor optimization of an assay, substantial amounts of resources and time would be wasted. For development of a next-generation PEV, a head-to-head comparison study with the new vaccine candidate and RTS,S or R21 vaccine can be performed. In such a study, any assays that showed CoP for RTS,S or R21 vaccines would be selected. However, such an approach cannot be utilized for BSV or TBV development at this moment, because there is no approved BSV or TBV. 

The general topics of malaria vaccine development and details for each of BSV and TBV current candidates and assays can be seen in recent reviews [4,5,6,7,8,9,10,11,12]. Therefore, in this manuscript, instead of summarizing the details of individual vaccine candidates or assays, I try to debate general issues and pitfalls for assays that have been or will be used for BSV/TBV vaccine development. More specifically, after reviewing several general key concepts of assays for vaccine development, I discuss important considerations during assay selection, standardization, qualification, validation, and interpretation of the assay results. Multiple assays that have been used for *P. falciparum* BSV or TBV development are cited as example cases in this review. However, it does not mean those assays are superior to non-cited assays. The best assay(s) could vary depending on the target molecule and usage of the assay results. Furthermore, not limited to *P. falciparum* BSV or TBV, similar assay development can be applied for any *P. falciparum* or *P. vivax* vaccines, as well as for any preventative or therapeutic monoclonal antibodies.

## 2. Correlate of Protection (CoP) vs. Surrogate of Protection (SoP)

Multiple terminologies, such as “correlate of protection (CoP)”, “surrogate of protection (SoP)”, or “immune marker of protection”, have been used by different researchers and regulatory agencies to explain the same concept, or more problematically, the same terminology could be used to explain different concepts, in several cases. The inconsistency makes it difficult to discuss assays for vaccine development. To avoid confusion for readers, I define “CoP” and “SoP” as follows in this review based on the definitions by the WHO [13]. However, different definitions could be used in different publications or guidance. I define “CoP” as “a statistically significant correlation between an immunological marker (e.g., vaccine-induced antibody titer or neutralizing activity) and a clinical protection measurement (e.g., time to first malaria episode or sterile protection in controlled human malaria infection (CHMI) trial)”. On the other hand, “SoP” is defined as “a statistically significant correlation between an immunological marker and a clinical protection measurement, and the assessed immunological mechanism has a direct link to the protection”. Based on these definitions, all SoP should be CoP, but SoP cannot be claimed until causality is confirmed. A single vaccine could have multiple CoP (i.e., results from each of multiple different assays correlate with a protection measurement) and/or multiple SoP (e.g., a vaccine needs both humoral and cellular immunity to induce protection). It is ideal to determine the SoP of a vaccine, but even the CoP of several licensed vaccines have not yet been established [14,15]. For licensed vaccines with known threshold values of protection, the values are almost always defined either by antibody titers or antibody neutralizing activity [14,15]. However, that fact does not necessary mean that the other immunological mechanisms, such as innate or cellular immunity, do not contribute to the protection (causality) induced by the vaccines. The licensed vaccines could have many other CoP or SoP, but such CoP/SoP might be practically more difficult to establish (e.g., difficult to collect test specimens and harder to obtain reproducible results). When a researcher selects an assay for vaccine development based on a preceding study(s), it is critical to evaluate whether the referenced study has demonstrated either CoP, SoP, mechanism of protection, or something else.

## 3. Reported CoP for RTS,S, R21, and Some BSV

Correlations between antibody titers and clinical protection have also been seen in RTS,S and R21 trials [16,17,18]. However, further investigations of the RTS,S vaccine have revealed that the IgG subclass [19], avidity [19,20], breadth of IgG response [21], and multiple functional humoral responses measured by system serology [22] are also CoP. Furthermore, correlations between many transcriptomic profiles [23] or antibody responses against off-targets (i.e., non-PfCSP antibodies) [24] and clinical protection have been reported. Recent Ph 2a [25] (vaccine efficacy is evaluated in a CHMI trial) and Ph 2b [26] (vaccine efficacy is evaluated in people who live in a malaria endemic area) clinical trials with anti-PfCSP monoclonal antibody (mAb) inoculations have clearly demonstrated that the antibody dose is SoP in the trials. However, the trials cannot answer whether the mAbs work by themselves, through antibody-mediated other immune mechanism(s) (e.g., antibody-dependent complement activation or phagocytosis), or a combination of both. In addition, mAb inoculation does not necessarily represent all immune mechanisms induced by RTS,S or R21 vaccinations. 

Once a CoP is established, determination of the threshold value of protection is the next step. However, in the case of malaria, there is no report of the threshold level of protection for either RTS,S or R21. In some of the abovementioned CoP studies for RTS,S, when the participants were divided into protective or non-protective groups, there were significant differences in the immunological readouts between the two groups. However, substantial overlap was observed between the two groups, e.g., some people with higher responses could be in the non-protective group, while other people with lower responses could be in the protective group [19,20,21,22,24,27]. If we cannot determine the threshold value, then a receiver operating characteristic curve analysis (at each of different levels of immunological measurement, determine the chance to correctly classify “protective” individuals as “protective” or misclassify “protective” individuals as “non-protective”), such as was done in one of aforementioned studies [22], will be beneficial for future vaccine development. 

To determine the CoP or SoP, a test vaccine must show clinical protection in a Ph 2a, 2b, or 3 trial. For BSV against *P. falciparum*, while some strain-specific effects (combination B [28] and AMA1 (apical membrane antigen 1) [29] vaccines) or preliminary protection effects that were evaluated in Ph 1b trials (MSP3 (merozoite surface protein 3) [30] and SE36 [31]) have been reported, only two vaccines, GMZ2 (fusion protein including *P. falciparum* glutamate-rich protein and MSP3) [32] and RH5 (reticulocyte-binding protein homolog 5) [33], have shown statistically significant protective effects in Ph 2 trials. Therefore, there is no established CoP for the other BSV candidates, other than CoP shown in a non-human primate (NHP) challenge model with *P. falciparum* inoculation for several BSV candidates, such as AMA1 and MSP1 [11]. For the GMZ2 vaccine, while vaccine-induced anti-GMZ antibodies demonstrated functional activities in an antibody-dependent cellular inhibition (ADCI) assay [34] and a bead-based phagocytosis assay [35], antibody titers measured by an enzyme linked immunosorbent assay (ELISA) have been the only CoP reported so far [35]. For the RH5 vaccines, both antibody titers and a functional activity measured by growth inhibition assay (GIA) correlated with in vivo protection in vaccinees after CHMI [33]. Furthermore, many other immunological parameters, such as antibody-dependent neutrophil phagocytosis, IgA1, binding to the neonatal Fc receptor, also correlated with in vivo protection based on a systems serology analysis [33]. Interestingly, the authors compared the results from the RH5 trial with their previous AMA1 Ph 2a trial [36], where some individuals in the AMA1 group demonstrated up to 20–30% inhibition in parasite multiplication (i.e., in vivo protection), even though, as a group, there was no significant difference between the AMA1 group and the control group in parasite growth in the trial. Regardless of the RH5 or AMA1 vaccines, individuals with the same level of in vitro GIA activity demonstrated a similar level of in vivo protection [33]. The results suggest that a similar CoP might be used both for RH5- and AMA1-based future vaccine development. An attempt to determine a mechanism of protection for an RH5 vaccine was conducted using a NHP challenge model with an anti-RH5 mAb that had functional activity determined by GIA [37]. The mAb had a mutated human IgG1 Fc region, which prevented engagement of the mAb with complement or Fc-receptor effector mechanisms but still demonstrated a protective effect in the challenge model. While the study clearly demonstrates that a direct effect (not antibody-mediated cellular immunity) of the anti-RH5 antibody can control parasite growth in the NHP model (SoP), there could be other SoP after RH5 immunization. 

For BSV against *P. vivax*, a significant protection was observed in a Ph 2a trial with a *P. vivax* Duffy-binding protein vaccine, and similar to RH5, both antibody titer and GIA activity were reported as CoP [38]. In addition, several cellular responses, such as the % of IFN-γ^+^ cells within CD4 memory T cells and % of antigen-specific cells within memory or plasma B cells, also correlated significantly with the in vivo protection [38,39].

For TBV, none of the vaccine candidates have yet demonstrated a reduction of transmission in a field study. Thus, no CoP (or SoP) can be determined. However, based on the mechanism of action, transmission-blocking activity (TBA; reduction in infected mosquitoes) or transmission-reducing activity (TRA; reduction in oocyst density) measured by a standard-membrane-feeding assay (SMFA) or direct-membrane-feeding assay (DMFA) are assumed to be SoP at this moment. Once a TBV reaches to a Ph 2 trial, direct skin feed assays (DSF) could be utilized, in addition to the SMFA or DMFA. When a TBV shows an effect in a field Ph 2b trial, the validity of SMFA/DMFA/DSF readouts as CoP/SoP should be revisited. 

## 4. Assay Standardization, Qualification, and Validation

There is also confusion in terminologies regarding this topic in the field. Several words, such as “standardization”, “optimization”, “qualification”, “pre-validation”, “validation”, or “partial validation”, have been used interchangeably in some cases but differently in other cases. Several guidelines have been published for assay validation, such as “Bioanalytical Method Validation, Guidance for Industry” by the U.S. Food and Drug Administration or “ICH Topic Q2(R1) Validation of Analytical Procedures; Text and Methodology” by the European Medicines Agency (EMA). In addition, an updated version of “ICH Q2(R2) guideline on validation of analytical procedures Step 5” by EMA will be effective from June 2024. On the other hand, there is no commonly accepted definition for “standardization”, “optimization”, “qualification”, or “pre-validation”. Not only is there confusion in the terminology, but there could also be different processes of “validation” for different assays, as each assay needs to be validated by “fit-for-purpose” [40]. In other words, depending on the characteristics and/or usage of the assay, different assays could be validated differently. Although assay validation is not required before a Ph 3 trial, those guidelines are helpful for researchers to consider which assay characteristic(s) should be evaluated, even when an assay is used for a preclinical study. In this review, I define “assay standardization” as a process to establish a detailed assay protocol (e.g., assay materials, a reference standard, and incubation condition for each step are specified); “assay qualification” as a process to measure one (or multiple) validation parameter(s) in a “standardized” assay; and “assay validation” as a process to confirm an observed assay result which matches with a predetermined parameter(s) in a “standardized” assay. For instance, in assay qualification, a researcher measures a variation (e.g., standard deviation (SD) or percent coefficient of variation (%CV)) in the observed results when the same sample is tested in multiple independent experiments as a part of “precision” evaluation. On the other hand, for assay validation, an accepted precision criteria should be predetermined (e.g., %CV should be <20), and an experiment will be conducted to check whether the observed results will match with the predetermined threshold (e.g., observed %CV = 10; thus, the assay is validated for the “precision” criteria). 

The major assay validation characteristics (e.g., specificity, sensitivity, and precision) described in the guidelines are summarized in Table 1 (depending on the guidelines, slightly different terminologies are used, but the basic concept is common). While all of them are important, “sensitivity”, “range”, and “precision” are the minimum characteristics that must be evaluated, even if an assay is used for the early stages of vaccine development (e.g., candidate discovery and preclinical studies). In other words, for each assay, the upper and lower limits need to be determined (“sensitivity”); then, the error of assay (EoA; how much variation in the signal is observed when the same sample is tested in different wells/plates/days or by different operators) should be evaluated (“precision”) within the upper and lower limits (“range”). This sounds obvious, however, only limited publications are available for assay qualification or validation in the malaria field. Based on the definitions above, there is no publication discussing assay validation, and only a few assays were qualified, such as ELISA for RTS,S [41], a mouse challenge model using transgenic *P. berghei* parasites expressing Pf CSP [27,42], GIA [43], and SMFA [44], and some additional studies have evaluated a part of assay precision (e.g., [45,46,47,48]). Thus, currently, it is very challenging to interpret published results by researchers from different laboratories. Let us consider a hypothetical scenario where a group of mice immunized with vaccine candidate A showed 20% mean inhibition in one study, and another vaccine candidate B group showed 30% mean inhibition in another study. In this scenario, if assay-to-assay variation of the reported assay could be as much as 15% in the mean inhibition, even when the two experiments were conducted by the same research group using the same assay, we cannot tell whether vaccine B is better than vaccine A. The two vaccines could be the same, or vaccine A might be better. If all the samples can be tested together in a single assay, assay-to-assay variation is not an important factor to consider. However, it is practically impossible to test all samples together in most cases. Comparisons to historical data within a laboratory and/or comparisons of data from different laboratories have been done widely, without knowing the sensitivity, range, and precision of the assay. There is a risk that a decision made by such comparisons could be wrong, thus decelerating vaccine development.

## 5. Species Selection for Preclinical Immunization Studies

Species selection is one of the vexing issues for vaccine development. Since it is practically impossible to compare all possible vaccine formulations or immunization strategies in humans or NHPs, early development studies are usually done using rodents. However, translatability of the rodent results to human studies has been questioned for a long time [49,50]. For drug or mAb development, dose per body weight (or dose per surface area) can be used to predict human doses from animal studies, but there is no such conversion formula for vaccines [51]. For example, there is a >100-fold difference in weight between mice (~30 μg or less) and children (~3 kg or more), but it is not rare to perform an animal immunization study using <10-fold difference in dosage compared to human dose (e.g., 5 μg/shot for the mouse and 10 μg/shot for the human). Therefore, it is very difficult to compare the quantity of immune responses (e.g., antibody titers) among different species even when the same vaccine is tested both in mice and humans. For the quality of the immune response (e.g., the amount of antibodies required to show the same level of inhibition in a functional assay), there are several studies to evaluate the species effect when different species are immunized with the same vaccines. In the studies, significant differences were observed between mice, rabbits, and humans for AMA1 and MSP1 vaccines judged by GIA [52] or for a Pfs25 vaccine judged by SMFA [53]. In the case of the Pfs25 vaccine, the difference in IC_50_ (anti-Pfs25-specific antibody concentration that gave 50% inhibition in SMFA) between rabbits and humans was as much as 20-fold (4.2 and 85.6 μg/mL, respectively) [53]. A similar large difference in quality was reported for another Pfs25 vaccine between mice and humans [54]. The quality difference between mouse and human might be explained, at least in part, by differences in the epitope recognition, affinity, or avidity of induced antibodies. To overcome this issue, several humanized transgenic mice, where human IgG loci are inserted to replace mouse IgG genes, such as Kymouse [55], have been developed. However, it is not clear whether such transgenic mice can completely mimic human immune responses for all vaccines (e.g., antigen presentation cells are still of mouse origin, not human dendritic cells), and usually, such transgenic mice are too expensive to use for large-scale screening. Above, GIA and SMFA were conducted without any complement or cells in the assays [52,53,54] but still showed significant differences in quality among different species. If a similar comparison is performed using another assay, where complement and/or cellular components are involved, differences in the Fc region of antibodies (e.g., IgG subclasses and affinity to human Fc receptors) may make it more difficult to predict human responses from animal models. Furthermore, a ranking of different vaccines could also differ among different species. When head-to-head comparison studies were performed in mice, NHP, and humans using the same Pfs25 and Pfs230 vaccines, while both vaccines were equally effective (judged by SMFA) in mice, the Pfs230 vaccine was superior to the Pfs25 vaccine in NHP and humans [56]. In general, NHP is considered as a better model than rodents to predict human responses, but there are noticeable obstacles, such as cost and ethical issues, to conducting large NHP studies. In addition, a recent study suggested that NHP might not be a perfect model. When a detailed antibody profiling was performed after controlled malaria infections in humans and *Aotus nancymaae* monkeys, while the IgG and IgM responses were similar overall, the IgA profiles were quite different between the two species [57]. Considering all the limitations, the current best approach is to interpret the animal results with caution. Once a vaccine candidate shows a protective effect in a human Ph 2 trial, not only CoP determination but also reevaluation of animal models for the specific vaccine will be beneficial. 

## 6. How to Select an Assay for Vaccine Development?

**Figure 1:**
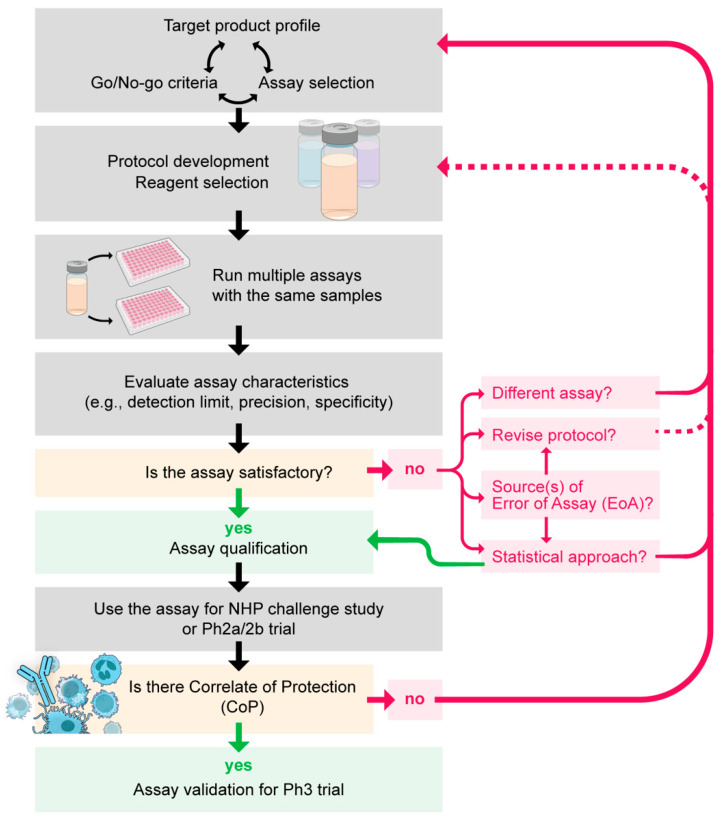
General flow from assay selection to validation. NHP, non-human primates; Ph 2a/2b trial, human phase 2a or 2b trial; Ph 3 trial, human phase 3 trial.

From here, I want to debate how to select, standardize, qualify, and validate an assay, and I then discuss general issues and pitfalls for the interpretation of assay results. Using several antibody-based assays as examples, I attempt to cover these topics as generally as possible below. However, each assay needs to be standardized/qualified/validated differently, depending on the characteristics and usage of the assay. Therefore, further consideration for assay-specific factor(s) may be required. The general flow from assay selection to validation is seen in Figure 1. 

Assay selection is closely linked with a target product profile (TPP) of a vaccine (what are the desired characteristics of a new vaccine, such as efficacy, cost, and stability) and Go/No-go criteria. For example, if an immunological TPP of a new vaccine is that the new vaccine should induce >30% better functional activity than an existing one, the developer needs to have an assay that can detect at least 30% difference. If the best available assay can detect only two-fold difference (>100% difference), then the TPP should be set accordingly. In addition, depending on the immunological Go/No-go criteria (e.g., what data trigger production of a recombinant protein for a Ph 1 study), assay selection or standardization may be relaxed or strict. For example, if an immunological Go/No-go criterion is that a vaccine can induce a detectable level of antigen-specific antibody by ELISA, it may not be required to perform standardization or qualification of a functional assay until the vaccine shows a protective effect in a Ph 2 trial. 

Another important consideration for assay selection is whether the assay of interest can be utilized from animal studies to a human clinical trial and can be done routinely in human vaccinees. Assuming a CoP by assay A is established in a Ph 2 trial, if the assay A cannot be used for animal immunization studies or cannot be done routinely in a human Ph 1 (or 2) trial, the value of assay A is limited from a vaccine development point of view, although the CoP result is interesting in terms of immunology and vaccinology. Recently, advanced immunological assessments, such as analysis of samples collected by hepatic fine needle aspiration [58] or bone marrow aspiration [59], have been done in human malaria vaccine trials, and such studies have provided detailed knowledge of human immune responses against the vaccine of interest. However, it is questionable whether such an approach can be done routinely in human trials and how to use the data in animal studies (e.g., can we use the same threshold of percent antigen-specific memory B cells in mice and humans to predict protective or non-protective?). Conversely, if an assay can be done only in mice but not with samples collected from a human trial, for example, a researcher needs to consider whether it is worthwhile to standardize/qualify the assay. 

Among functional assays for BSV, the GIA has been the most widely used assay [10,11,12], assuming the GIA result could be the SoP for the target antigens. In the majority of cases, GIA is used to evaluate the functional activity of antibodies against merozoite antigens (e.g., AMA1, MSP1, and erythrocyte-binding antigen 175), but antibodies that target the late trophozoite or schizont stages of blood-stage parasites were also evaluated by GIA (*P. falciparum* schizont egress antigen-1 [59] and *P. falciparum* glutamic-acid-rich protein [60]). However, not all BSV candidates induced GIA-positive antibodies, and some vaccine candidates, such as VAR2CSA-based vaccines [61], are expected to work by other mechanisms (e.g., prevent the binding of infected red blood cells to human endothelial cells), not through a GIA mechanism. Furthermore, a single vaccine could have multiple SoP. Thus, other assays that have demonstrated CoP in epidemiology studies, such as ADCI [46,62], opsonic phagocytosis [63,64,65,66], antibody-dependent respiratory burst (ADRB) [67,68,69], and complement fixing by antibody [70,71,72], have also been used for BSV development. While such research for immunity induced by natural infection is unarguably important, the results should be interpreted carefully when an assay(s) is selected for vaccine development. First, natural infections induce multiple immune responses against multiple antigens. In several immune epidemiology studies, many CoP were reported for multiple antigens within a single cohort study, and multiple immunological readouts also correlated with each other [73,74,75,76,77,78,79]. In some studies, the functional evaluation of affinity-purified antigen-specific IgGs or multiple linear regression analysis have been conducted to evaluate antigen-specific responses. However, even when an antigen-specific immune response is proved to be CoP in an epidemiology study, there is no guarantee that the antigen-specific responses caused by >1000 antigens (natural infection) are the same as those by a single or a few antigens in vaccination. Furthermore, meta-analyses have shown that CoP could vary among different epidemiology studies, even when the same assays were employed [80,81,82]. The discrepancy could be explained by differences in assay protocol (e.g., reagents and assay conditions); by negative interactions (interferences) between different antibodies induced by natural infection [11,83]; or by many other factors that vary among different epidemiology studies, such as age, endemicity, and parasite strains. Thus, the selection of assay based on a single epidemiological study should be done carefully. Several non-GIA assays have been utilized for human clinical trials, and the vaccine-induced antibodies showed positive responses in the studies, such as ADCI (for MSP2 [84], MSP3 [85], GMZ2 [34,35], SE36 [86], and P27A [87] vaccines); opsonic phagocytosis (GMZ2 [35]); ADRB (MSP1 [88]); complement fixation (MSP2 [72]); or infected red blood cell binding inhibition to chondroitin sulfate A (VAR2CSA [89,90]). The principles and characteristics of these assays are summarized in Table 2. While the positive results in the human trials support the usage of these assays for vaccine development, the fact does not necessarily mean that the assay measurement can be CoP (or eventually SoP). Similarly, there is no human evidence that the GIA readout can be CoP for non-RH5 vaccines at this moment. Thus, as far as resources allow, it is better to select many assays (including other assays that are not described above) based on the predicted mechanism of action for a target antigen, until a CoP has been determined for the target antigen in a Ph 2 trial. 

For TBV, SMFA and DMFA are the two major assays for early-stage vaccine development. However, both SMFA and DMFA are low-throughput assays; thus, other assays, such as the *P. falciparum* dual gamete formation assay [91] or *P. berghei* ookinete development assay [92], have been used for the mass screening of transmission-blocking drugs. However, in those drug mass screening studies, the final confirmation of selected drugs was conducted by SMFA. Therefore, at this moment, while researchers need to select SMFA, DMFA, or both, depending on the malaria species (either *P. falciparum* or *P. vivax*), antigen, or TPP, there is no alternative approach for the early stage of *P. falciparum* or *P. vivax* TBV development. The characteristics of these assays are summarized in Table 3.

## 7. How to Standardize, Qualify, and Validate an Assay?

Once an assay(s) is selected, an assay protocol should be developed, and a published method can be used as a reference if available. However, establishment of negative and positive controls for the specific assay may not be so easy in some cases. The positive control should give a positive signal in every experiment by definition, and the positive control is likely to be required to demonstrate a dose–response in the selected assay (i.e., higher concentration gives higher signal and vice versa). It might be an option to develop a qualitative assay (i.e., assay result is binary, yes or no), not a quantitative assay. However, considering a possible difference in threshold values among different species and the final goal of assay development (i.e., establishment of CoP in humans later), a qualitative assay has a higher risk of failure (i.e., the assay turns out to be useless) than a quantitative assay in general. Selection of a negative control is also not so straightforward. First, there are different types of negative controls. For example, a negative control for an assay that evaluates an activity of mouse sera could be an assay buffer, normal mouse serum, serum collected from mice immunized with an adjuvant alone, or serum collected from mice immunized with non-malaria antigen with an adjuvant. The last one is the ideal, but such serum (or antibody) may not be available in enough quantity. In addition, to determine the lowest limit of assay, usually, multiple different negative controls are required. If sufficient positive and negative controls are not available in the early stages of vaccine development, it might be wise to switch to another assay(s). 

After the initial assay development, once a (tentative) assay protocol is set, a set of samples with various activities (or serial dilutions of a positive control) are tested repeatedly in multiple independent assays to check the assay validation characteristics (Table 1). It is obvious that the set of samples should cover from the lowest to the highest qualification (or detection) limit of the assay, but there is no consensus how many repeats at how many different levels of activity need to be tested for assay standardization or qualification. Based on the EMA’s guidelines for assay validation, a minimum of three replicates at three to five different levels are recommended to evaluate the precision or accuracy in general (the number could vary depending on the type of assay). However, the goal of assay validation is to demonstrate that the assay can behave as predicted. To determine the “normal behavior” of an assay (the goal of assay qualification), more samples and more experiments are required in general, and more data are always better from a mathematical point of view. If the number of samples or repeat assays is too few at this stage, there is a risk to underestimate the EoA or limits of the assay. If so, interpretation of the assay results may not be accurate, and there is a higher risk of failure during assay validation (e.g., results from validation experiments show a higher variation than the predetermined acceptance criteria). Having said that, practicality is also an important factor to consider. Based on my experience, I recommend performing five repeat experiments using five to seven different samples with different activities (or five to seven different dilutions of a single sample) per experiment to obtain a reasonable estimate for the assay characteristics if possible. 

Once the initial data are obtained, based on the usage of the assay (e.g., what level of precision is desired within “range” of the target assay), a researcher decides whether the current assay is satisfactory or not. If the answer is yes, then the assay is qualified using the values observed in the above experiments. On the other hand, if the answer is no, depending on which characteristic(s) is problematic, different approaches need to be taken. For example, if there is an issue for specificity or selectivity, test samples might be diluted with an optimal buffer or purified IgG used, instead of the serum. Another example is, if there is an issue in stability, fresh (not freeze–thaw) samples might be used for the assay. When an assay is not satisfactory at this stage, in many cases, the issue is related to the precision characteristics. Thus, the topic is discussed in detail in the next two paragraphs. 

Based on “Bioanalytical Method Validation, Guidance for Industry” by the U.S. Food and Drug Administration, the accepted criteria for precision are ±15%CV (coefficient of variation) for a chromatographic assay and ±20%CV for ligand-binding assays. Therefore, unless a researcher needs to detect a very minor difference, a 15–20%CV level of EoA is considered acceptable. However, in some assays, such as GIA, a constant SD (standard deviation) model is better than a constant CV model to express the EoA [43]. Thus, as an indicator of precision, different parameters, such as %CV, SD, or interquartile range, might be compared to identify the best one for a specific assay. In a complicated biological assay, such as one involving immune cells (e.g., ADCI) or mosquitoes (e.g., SMFA), it might be very challenging to reach a desired level of precision, regardless of the precision indicator selected. If the EoA in the initial experimental data set is too large, as a first step, a source(s) of EoA needs to be determined using a “balanced” study design. The “balanced” design in this context means a study design that allows an evaluation of the contribution of each of two (or more) potential sources of EoA. For example, if one wants to determine whether the EoA comes from cell-to-cell variation or from day-to-day variation, multiple batches of cells need to be tested on each assay day, and the same experiment should be repeated on multiple days. If different batches of cells are used on different assay days, it is difficult to separate out cell-to-cell and day-to-day variations. To perform additional “balanced” experiment(s) just to optimize an assay may sound like a waste of resources, especially for a researcher who has not worked on vaccine development before. However, this could be the clearest way to reach a correct conclusion. Depending on the size of the EoA, the day-to-day variation may need to be further differentiated into well-to-well, plate-to-plate, and/or operator-to-operator variations. Only a few publications discuss cell-to-cell variations in assay readouts, but when it is assessed, a significant impact of primary human cells on EoA have been reported for ADCI [46] and ADRB [47]. In the ADCI study, besides a comparison among different donors, monocytes were collected from the same donors on different days. Up to a 40% difference in the specific growth inhibition (SGI) readout was observed on different days when monocytes from the same donors were used (while it was not clear how much difference came from the monocyte-to-monocyte variation and how much from the day-to-day variation). To avoid such issues with primary cells, cell lines (e.g., THP-1) can be used in some assays [93,94,95]. However, switching to cell line(s) may not be so easy for some assays, as available cell lines might not be identical to the primary cells. For example, THP-1 cells do not express the FcγRIII receptor [96]. Not only the batch of white blood cells but also the batch of red blood cells (RBCs) could affect an assay result. Based on a recent study, the RBC-to-RBC variation is ~four times bigger than the day-to-day variation in the % inhibition readout of GIA [43], and there is no mature RBC cell line to replace the primary RBC. 

In an ideal scenario, after determination of the source of EoA, the assay protocol is optimized further to reduce the EoA as desired. However, protocol modification might be practically impossible (e.g., maintain *P. falciparum* in non-RBC cell line), or the EoA could still be unacceptably large after the modification. If this is the case, there are three options (or a combination of the last two) to go: (i) switch to another assay, (ii) change the Go/No-go criteria (or TTP), or (iii) the statistical approach. Option (i) is simple, but whether or not there is an alternative and better assay is another question. Option (ii) is to change the target based on the EoA so that Go/No-go criteria can be measurable. For example, it is known to be difficult to measure lower % inhibition values accurately in SMFA (especially <60%TRA (percent inhibition in TRA)), regardless of laboratories performing the assay [97], unless impractically large numbers of mosquitoes are examined [45] or the same sample is tested in multiple assays. Therefore, if SMFA results (from a single assay) are used for one of the Go/No-go criteria, a Go criterion of “30% of vaccinees should show >80%TRA” (which is easier to measure accurately) is more appropriate than another criterion of “50% of vaccinees show >50%TRA” (which is hard to measure), for example, based on the assay characteristics. Option (iii) has been used for several studies. One such approach is to perform three independent assays using three different batches of cells, then to report an average (median or geometric mean) from the three assays as the final results (e.g., [47,93,97]), assuming batch-to-batch variation is random. This is easy to understand, but this approach requires three times more resources than a single assay. Therefore, when the number of samples is large, it could be difficult to execute. Another approach for option (iii) is to present 95% confidence intervals (95% CIs) of an observed value (e.g., [97,98]). By doing so, it is easier to intuitively estimate whether or not two (or more) observed values are truly different. For example, one sample that has 50% inhibitory activity with 95% CI of 30 to 70 is not likely to be significantly different from another sample that has 65% inhibitory activity with 95% CI of 50 to 80. However, if the data do not follow a Poisson distribution (or any other common distribution where a method to calculate the 95% CI is known), it might be necessary to build an assay-specific mathematical model to estimate the 95% CI. Since all the three options have pros and cons, the best option should be selected on a case-by-case basis. 

The “robustness” characteristic is one of the complicated characteristics to evaluate, even though the concept is simple: how much variation in the analytical procedure is allowed to obtain the same signal when the same sample is tested. For the sake of discussion, let us imagine an ELISA protocol where there are three incubation steps (primary and secondary antibodies and substrate). If the final protocol is set as “primary antibody is incubated for 60 ± 5 min at room temperature”, for the robustness evaluation, a researcher needs to prove (at a minimum) that the resulting ELISA titers are the same at 55, 60, and 65 min and at 20 and 25 °C (assuming room temperature means 20–25 °C), i.e., six different conditions will be tested. On the other hand, if one develops a stricter protocol as the “primary antibody is incubated for 60 min at 25 °C”, then, no robustness test is required for this step. However, if an actual experiment is done for 61 min or at 24 °C by mistake, it is considered as a protocol deviation. There are two more incubation steps in the ELISA, and there could be other factors for which an accepted window needs to be determined (e.g., dilution factor of a secondary antibody might be optimized by a specific batch of product). While it is simple to evaluate each experimental parameter, it is very difficult to judge how many (and how deep) parameters should be experimentally assessed. More complicated assays, such as ADCI and DMFA, have more parameters to evaluate than ELISA, and the importance of each parameter could vary depending on the assay (e.g., a small deviation in incubation temperature may be critical for one step but not for the other steps). Therefore, it might be practically impossible to evaluate each of the parameters in an assay. Having said that, a robustness test for a key parameter of an assay may be helpful to identify the source of EoA. If the assay characteristics are not satisfactory at this stage, the robustness aspect of the assay could be investigated further. On the other hand, if satisfactory, a detailed evaluation of the robustness may be performed later, as it is not clear whether the assay is helpful for vaccine development until it is used for a Ph 2 trial (or a NHP challenge study).

Once the results of a qualified assay are shown to be CoP in a Ph 2 trial, the next step is assay validation before a Ph 3 trial. As far as the assay has been qualified, the accepted criteria of the assay validation can be set easily based on either the several validation guidelines or the results of assay qualification. However, a researcher might be better off discussing this with a regulatory agency on a case-by-case basis (e.g., whether their validation criteria is reasonable when acceptable criteria is larger than 15–20%CV or how much robustness tests should be conducted). 

## 8. How to Interpret and Report the Results of an Assay?

While it sounds easy to interpret or report assay results, there are several common issues and pitfalls that make harder to compare results. Appropriate analysis and reporting the results will help researchers compare data from their own laboratories and/or from different laboratories. The first point to consider is whether the results are better analyzed (and reported) with the original numbers, transformed numbers (e.g., log-transformation), or relative ratios (e.g., fold-change or % inhibition). A rational decision should be made based on an expected mechanism of action and a mathematical justification, but in several cases, the decision is made based on how people published in the past, while some of the analysis may no longer be appropriate based on the current knowledge. 

When original numbers do not follow a Poisson distribution, a log-transformation is one of the commonly used methods, such as analyzing antibody titer data, and it is the first and easiest step to try. However, if the log-transformation turns out to be not ideal, then other transformations (e.g., inverse or square root) should be evaluated, and there are mathematical techniques to predict the best transformation (e.g., Box-Cox transformation). 

In addition to the transformation of observed values, it is also common to report the results in relative ratios. For example, a researcher may report a relative response against a control, % inhibition, or a more complicated way (e.g., SGI used for ADCI). These are common, but the potential risks for such an analysis are rarely discussed in the literature. An issue for the relative ratio analysis is that completely different data sets could give the same ratio. For example, a change from 100 events to 1 event and that from 100,000 events and 1000 events are both 100-fold reductions. Therefore, it is important to evaluate whether or not the two changes have the same biological meaning in the assay. This type of issue was also seen in several clinical trial publications. Suppose there are two vaccinees, volunteers A and B, who received the same vaccine. Volunteer A shows a titer of 1 at baseline, then 100 titers after a vaccination, while volunteer B shows 1000 and 100,000 titers, respectively. Since the baseline titers are different between the two individuals (due to preexisting immunity induced by natural infections), the antibody results are analyzed using a fold-change readout, instead of actual titers, in some studies. Again, in this example, both individuals show the same 100-fold increase by the vaccination. However, if the antibody titers are CoP of the vaccine, it is reasonable to predict that volunteer B is more likely to be protected than volunteer A. Therefore, using fold-change as a readout to compare individuals or groups is questionable, unless there is a reason to predict that the fold-change could be a better CoP than actual titers. 

Regarding the % inhibition readout, it is usually calculated as 100 × (1 − Te/Co), where Te is a value from a test sample, and Co is a value of an assay control. Therefore, the % inhibition readout is one of transformations of the relative ratio readout, and thus, the same problem described above applies to % inhibition analysis as well. In addition, the formula for the % inhibition calculation could also cause another problem. For SMFA (and DMFA), it is becoming a consensus that a <50–60%TRA value may not mean a true inhibitory activity (i.e., within a range of EoA) if the sample is tested in a single assay with 20–40 mosquitoes (per group). However, this type of threshold is not set for “transmission enhancement (TE)”, and different definitions of TE have been used in multiple publications (e.g., [99,100,101,102,103]). Assuming the two samples A and B are tested by SMFA, and the mean number of oocysts are 10 and 5, respectively, when the %TRA of sample B against sample A is calculated, the value is 50 (= (1 − 5/10) × 100). On the other hand, the %TRA of sample A against sample B is −100 (= (1 − 10/5) × 100). In this case, the 50%TRA of sample B is not likely to be considered as a true inhibitory activity. However, sample A could be reported to have a true TE effect against sample B, because a value of “−100” looks to be a bigger effect than “50”, although both data show the same phenomenon, i.e., there is a two-fold difference in oocyst density between the two samples. If a researcher uses an 80%TRA cut-off (five-fold difference between the test and control samples) to define a “true” inhibition, then only <−400%TRA should be considered as a “true” TE. Not only for SMFA/DMFA, any negative value in % inhibition (i.e., “enhancement”) should be interpreted carefully, because there is the same issue in % inhibition calculation regardless of the characteristics of the assay. Talking about the misinterpretation of “enhancement”, it is also important to keep in mind that all assays have EoA; no assay is perfect. When many samples, all of which have true 0% inhibitory activities, are tested (or when a sample with true 0% inhibitory activity is tested in multiple assays), around half of samples (or assays) show “enhancement”, and the other half show “inhibition” when 0% inhibition is used as a cut-off value (and a small proportion of data could show exactly 0% inhibition by chance). Therefore, unless the “enhancement” threshold is set considering the EoA, even if 30–40% of test samples show an “enhancement” effect, it may not mean anything. This is also true for the “inhibition” results, but usually, researchers are more cautious in “inhibition” judgements. 

Another important point to consider when reporting an assay readout is whether the EoA is constant or not throughout the range of the assay. Multiple SMFA and DMFA studies have shown that the oocysts data cannot be explained by a Poisson distribution, and a variation (e.g., SD) of the oocyst number within a group is bigger when the mean oocyst of the group is larger, regardless of the species of parasites and mosquitoes used for the assays or laboratories [44,45,97,103,104,105]. Thus, lower %TRA values (i.e., higher mean oocysts in the test group) have a larger uncertainty than higher %TRA values. However, when %TRA values are presented without showing the uncertainty (e.g., with 95% CI), a general audience cannot interpret the results accurately. For example, a difference between 10 and 60%TRA looks bigger than another difference between 70 and 95%TRA, but the former can be statistically insignificant, while the latter can be significant. Another example is the GIA_50_ readout (concentration of antibody that gives 50% inhibition in GIA), where the EoA is bigger when the GIA_50_ value is larger [43]. If the EoA varies based on the level of signal, it is essential to present the data with the error range (e.g., 95% CI) or to transform the results so that EoA becomes constant.

Finally, to report the assay results, it is common to perform a statistical test to compare two or more groups. Nowadays, a misuse of a parametric test for analyzing data that have non-normal distributions is rare; however, a non-parametric test is not always the best choice. For example, a non-parametric test, such as a Wilcoxon–Mann–Whitney (WMW) test, is commonly used to analyze SMFA (or DMFA) data in many publications, but there is a risk to using the MWM test. Even a non-parametric test has important assumptions that affect its validity. Chief among these assumptions is the exchangeability that is achieved with independent observations. In other words, the exchangeability assumption is violated if SMFA data are handled without accounting for the dependence between observations within a feed (i.e., by ignoring within-feed correlations, simply summed oocyst data from multiple assays). One way to handle the data dependence is to explicitly model it. The oocyst distribution within a group of mosquitoes that are fed the same blood meal in SMFA can be explained by a zero-inflated negative binomial model with random effects to account for dependent observations [44,45]. To evaluate the issue of the WMW test mathematically, a simulation study was conducted using oocyst data generated by a SMFA-specific zero-inflated negative binomial random effects model published previously [44]. 

**Figure 2:**
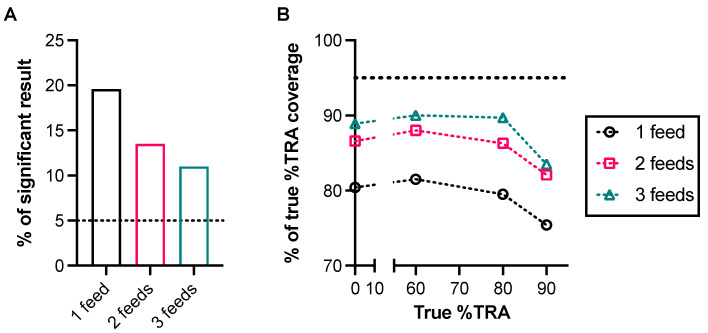
Accuracy of the Wilcoxon–Mann–Whitney (WMW) test for standard membrane feeding assay (SMFA) data. A simulation study was conducted using oocyst data generated by a SMFA-specific zero-inflated negative binomial (ZINB) model [44]. (**A**) In the first simulation, the true %TRA of a test sample was set to zero %TRA (i.e., no inhibition), and oocyst data for the control and test groups (20 mosquitoes per group per feed) were generated in three different scenarios where they were tested in 1, 2, or 3 independent feeding experiments. When the SMFA was conducted once (1 feed, n = 20 per group), the oocyst data of the test and control groups were compared by a WMW test. For the oocysts data from 2 or 3 feeds, the oocyst data from multiple feeds were simply combined, ignoring feed information (n = 40 for 2 feeds and n = 60 for 3 feeds per group), and then, a WMW test was conducted. The whole process was repeated 1000 times (1000 iterations), and a proportion of iterations where the WMW tests gave significant results for the oocyst data was calculated. Three additional simulations were performed for 3 different test samples, where the true %TRA activities of them were set to 60, 80, or 90 instead of zero in the first simulation. Same as before: in each simulation, three different scenarios, i.e., the sample was tested in 1, 2, or 3 feeds, were evaluated. (**B**) Using the oocyst data generated as previously described, a 95% CI of %TRA in each scenario in each iteration was estimated using an asymptotic Hodges–Lehmann approximation based on the Wilcoxon test. For each scenario in each simulation, a proportion of iterations (out of 1000 iterations) where the estimated 95% CI from the oocyst data covered the true %TRA value was calculated. The horizontal dotted lines show target (5% or 95%) values if the tests and CIs are working correctly. When the simulation data were analyzed by the ZINB model, the type I error rate and the 95% CI coverage rate were ~5% and ~95%, respectively, in all scenarios as predicted.

In the simulation, four different levels of samples (true %TRA = 0, 60, 80, and 90) were tested in one, two, or three independent assays; then, oocysts data from two to three assays were simply summed, as done in several published studies. In each iteration, using the oocyst data, two evaluations were conducted: whether (1) a WMW test gave a significant result (either inhibition or enhancement) and (2) 95% CI (determined by an asymptotic Hodges–Lehmann approximation based on the Wilcoxon test) covered the true %TRA levels of the test sample; then, the same process was repeated 1000 times. When true %TRA = 80 or 90, a WMW test gave “significant” results with >95% provability (as we hoped), even when SMFA was done once, and at true %TRA = 60, three independent feeds were required to show >95% provability of a “significant” call, which is not surprising, as a lower %TRA is difficult to detect in SMFA, as discussed before. However, at the true %TRA = 0 level (i.e., no activity), when only one feeding experiment was performed, the WMW test misdiagnosed (i.e., WMW test shows a “significant” effect, while it should not) in ~20% of cases, while the rate was supposed to be ~5% (Figure 2A). The rate of misdiagnosis became lower with more feeds, but even with three feeds, the rate was still 11%. For the coverage analysis (Figure 2B), while more feeds were better in general, even with three feeds, the 95% CI coverage rate was <90%, regardless of the %TRA level, while it should be ~95%. This simulation shows the importance of a proper selection of statistical test to reduce the risk of misinterpretation of the results. 

## 9. Conclusions

The results from immunological assays have been and will be utilized as a part of the Go/No-go criteria in vaccine development. Immunological readout is not the solo factor to determine a vaccine design; other factors, such as cost, scalability, and stability, are equally important. However, if the immunological decision is made based on an assay where uncertainty (error range) of the results is not evaluated systematically, such a decision has a higher chance to waste resources in the future compared to a decision based on a qualified (or validated) assay. In addition, a lack of uncertainty information in assay results makes it extremely difficult to compare data generated from different laboratories or even data from the same laboratory but performed in different experiments. In the case of COVID-19 vaccines, antibody titers and antibody neutralizing activity have been proven to be CoP in humans after vaccinations (at least during the first 5–6 months after immunization, where antibody titers/activities are relatively high) within 2 years of the COVID-19 pandemic emergence [106,107,108,109]. Furthermore, the WHO established the first international standard of anti-SARS-CoV2 immunoglobulin, by which all COVID-19 vaccine developers in the world could calibrate their binding and neutralization assays, in December 2020 within 1 year of the emergence [110]. Unfortunately, however, it is very unlikely that malaria vaccine development can follow the same path as done for COVID-19 vaccines, and each laboratory should establish a specific assay(s) based on the characteristics of the target molecule using their own standard(s). This review supports the establishment of a qualified (or ultimately validated) assay and guides a proper reporting of animal and human trial results, making it easier for the malaria research community to use the precious information obtained from the trials. 

## Figures and Tables

**Table 1:** Characteristics for assay validation.

Characteristics	Example of Interpretation ^1^
Specificity	Whether we can detect a signal of test antibody in the presence of an unrelated substance that may be expected to be present in a test sample
Selectivity	Whether we do not detect any signal when an analyte contains no specific antibody
Sensitivity	How low (or high) we can detect a signal of test antibody
Detection Limit	The lowest (or the highest) signal can be detected (but not necessarily quantitated)
Quantitation Limit	The lowest (or the highest) signal can be quantitatively detected
Range	The interval between the upper and lower levels of signals in which the analytical procedure has a suitable level of the other parameters
Precision	How much variation in a signal is observed when the same antibody is tested repeatedly
Repeatability	Intra-assay variability
Intermediated Precision	Inter-assay variability
Reproducibility	Inter-laboratory variability
Stability	Whether we can detect the same signal when a test antibody is kept at a storage temperature and/or after freeze–thaw
Dilution effects	Whether we can obtain the same result (a signal multiple by a dilution factor) when a test sample is diluted (but still within a “range” of assays)
Linearity	Whether (a transformation of) a signal of a test antibody is directly proportional to (a transformation of) the concentration of test antibody
Accuracy	Agreement between a conventional true value and an observed value
Robustness	How much variation in the analytical procedure (e.g., incubation time and temperature) is allowed to detect the same signal for the same sample

^1^ This table shows an example of interpretation for each validation characteristic, assuming the assay is an antibody-based assay. The interpretation can vary depending on the assay (and usage of the assay results).

**Table 2:** Functional assays used in BSV human clinical trials.

Assay	Principle	Characteristics
Growth inhibition assay (GIA) or invasion inhibition assay (IIA)	A test antibody is incubated with cultured parasites to determine the direct killing or invasion blocking effect of the antibody.	Most widely used assay for BSV development CoP in humans for a RH5-based vaccine Significant batch effect of RBC No measurement for cellular (or cell-mediated) immunity
Antibody-dependent cellular inhibition (ADCI) assay	A test antibody is incubated with human monocytes and cultured parasites to determine the parasite-killing effect by the monocytes.	Purified human IgGs that showed parasite clearance effects in a passive transfer study in humans had ADCI activity Significant batch effect of monocytes Only a few laboratories can perform the assay
Opsonic phagocytosis assay	A test antibody is incubated with parasites first; then, the opsonized parasites are further incubated with cells. A proportion of cells that internalize the parasites is measured.	Different studies use different protocols (e.g., purified merozoites, infected RBC, or antigen-coated beads for opsonization; THP1 cell line, purified monocytes or neutrophils, or whole PBMC for phagocytosis) Significant batch effect of monocytes/neutrophils Limited usage in human clinical trials so far
Antibody dependent respiratory burst (ADRB) assay	A test antibody is incubated with purified merozoites first; then, the opsonized merozoites are further incubated with neutrophils. ROS released from the neutrophils are measured.	Significant batch effect of neutrophils Limited usage in human clinical trials so far
Complement fixation assay	A test serum/plasma is incubated with purified merozoites; then, the amount of C1q that binds to the merozoite-specific antibodies is measured.	A high-throughput assay compared to the other assays in this table Instead of purified merozoites, a recombinant protein can be used Limited usage in human clinical trials so far
Infected RBC binding inhibition assay	A test antibody is incubated with infected RBC and CSA to determine how much binding between infected RBC and CSA is interfered with by the antibody.	A similar assay can be done for other PfEMP1-based vaccines. But only VAR2CSA-based vaccines have reached human clinical trials so far

RBC, red blood cell; PBMC; peripheral blood mononuclear cells; ROS, reactive oxygen species; CSA, chondroitin sulfate A; PfEMP1, *P. falciparum* erythrocyte membrane protein 1.

**Table 3:** Functional assays used in TBV human clinical trials.

Assay	Principle	Characteristics
Standard-membrane-feeding assay (SMFA)	A mixture of a test antibody and in vitro cultured gametocytes is fed to mosquitoes; then, a reduction in oocysts (intensity or prevalence) in the mosquitoes is determined.	Assay can be performed in a non-malaria endemic area Failure rate of assay (i.e., oocyst intensity/prevalence in a control group is too low to evaluate the efficacy of a test antibody) is relatively lower compared to DMFA/DSF Only a limited number of parasite strains can be used
Direct-membrane-feeding assay (DMFA)	Similar to SMFA but uses gametocytes in blood from an infected human (or monkey), instead of cultured gametocytes in SMFA.	Multiple strains of parasites can be used Infected human (or monkey) with high gametocytemia is required
Direct skin feed assays (DSF)	Mosquitoes feed directly on the skin of a vaccinated human.	Closest to a natural infection Multiple strains of parasites can be used Cannot be done before a Ph 1b or Ph 2 trial Failure rate of the assay is extremely high

## Data Availability

Not applicable.
